# Long COVID is associated with severe cognitive slowing: a multicentre cross-sectional study

**DOI:** 10.1016/j.eclinm.2024.102434

**Published:** 2024-01-25

**Authors:** Sijia Zhao, Eva Maria Martin, Philipp A. Reuken, Anna Scholcz, Akke Ganse-Dumrath, Annie Srowig, Isabelle Utech, Valeska Kozik, Monique Radscheidt, Stefan Brodoehl, Andreas Stallmach, Matthias Schwab, Emily Fraser, Kathrin Finke, Masud Husain

**Affiliations:** a Department of Experimental Psychology, University of Oxford, Oxford, OX2 6GG, UK; b Department of Neurology, Jena University Hospital, Jena, Germany; c Department of Internal Medicine IV (Gastroenterology, Hepatology and Infectious Diseases), Jena University Hospital, Jena, Germany; d Nuffield Department of Clinical Neurosciences, University of Oxford, Oxford, OX3 9DU, UK; e Center for Sepsis Control and Care (CSCC), Jena University Hospital, Jena, Germany; f Oxfordshire Post-COVID Assessment Clinic, Oxford University Hospitals Foundation NHS Trust, Oxford, UK; g Department of Psychology, LMU Munich, Munich, Germany

**Keywords:** COVID-19, Post-COVID conditions, Post-acute COVID syndrome, Cognition, Response time, Processing speed

## Abstract

**Background:**

COVID-19 survivors may experience a wide range of chronic cognitive symptoms for months or years as part of post-COVID-19 conditions (PCC). To date, there is no definitive objective cognitive marker for PCC. We hypothesised that a key common deficit in people with PCC might be generalised cognitive slowing.

**Methods:**

To examine cognitive slowing, patients with PCC completed two short web-based cognitive tasks, Simple Reaction Time (SRT) and Number Vigilance Test (NVT). 270 patients diagnosed with PCC at two different clinics in UK and Germany were compared to two control groups: individuals who contracted COVID-19 before but did not experience PCC after recovery (No-PCC group) and uninfected individuals (No-COVID group). All patients with PCC completed the study between May 18, 2021 and July 4, 2023 in Jena University Hospital, Jena, Germany and Long COVID clinic, Oxford, UK.

**Findings:**

We identified pronounced cognitive slowing in patients with PCC, which distinguished them from age-matched healthy individuals who previously had symptomatic COVID-19 but did not manifest PCC. Cognitive slowing was evident even on a 30-s task measuring simple reaction time (SRT), with patients with PCC responding to stimuli ∼3 standard deviations slower than healthy controls. 53.5% of patients with PCC's response speed was slower than 2 standard deviations from the control mean, indicating a high prevalence of cognitive slowing in PCC. This finding was replicated across two clinic samples in Germany and the UK. Comorbidities such as fatigue, depression, anxiety, sleep disturbance, and post-traumatic stress disorder did not account for the extent of cognitive slowing in patients with PCC. Furthermore, cognitive slowing on the SRT was highly correlated with the poor performance of patients with PCC on the NVT measure of sustained attention.

**Interpretation:**

Together, these results robustly demonstrate pronounced cognitive slowing in people with PCC, which distinguishes them from age-matched healthy individuals who previously had symptomatic COVID-19 but did not manifest PCC. This might be an important factor contributing to some of the cognitive impairments reported in patients with PCC.

**Funding:**

10.13039/100010269Wellcome Trust (206330/Z/17/Z), NIHR Oxford Health Biomedical Research Centre, the Thüringer Aufbaubank (2021 FGI 0060), German Forschungsgemeinschaft (DFG, FI 1424/2-1) and the 10.13039/100010661Horizon 2020 Framework Programme of the 10.13039/501100000780European Union (ITN SmartAge, H2020-MSCA-ITN-2019-859890).

**Research in context**

**Evidence before this study**

We searched Google Scholar and PubMed for original research or review articles about cognitive impairment after COVID-19, published up to 3 November 2023. We used terms relating to COVID-19 (SARS-CoV-2, influenza), post-acute symptoms (long COVID, post-COVID conditions, Post-Acute COVID Syndrome) and cognitive impairment (brain fog, cognitive deficit). Previous studies have shown that some people who recovered from the acute symptoms of COVID-19 might nevertheless experience deficits across an array of cognitive functions, including sustained attention, cognitive flexibility, and memory. However, most reports lacked consensus on the precise definition of post-COVID conditions and a common cognitive signature of post-COVID conditions remains unknown.

**Added value of this study**

In this investigation, we identified moderate to severe cognitive slowing in most patients with PCC, but not in most people who previously had COVID without developing PCC. This was replicated across two post-COVID clinics in Germany and the UK. To our knowledge, this is the first robust demonstration of cognitive slowing as a cognitive signature of post-COVID conditions.

**Implications of all the available evidence**

Using a 30-s web-based, self-administered psychomotor task, cognitive slowing in PCC can be reliably and easily measured as part of diagnostic work-up, and has potential to be a biomarker to track the progress of rehabilitation of PCC. To encourage researchers and clinicians to employ this task, we have ensured that it is available online with online feedback and all of our code is publicly accessible.

## Introduction

Post-COVID-19 condition (PCC), often known as “long COVID”, is a constellation of chronic symptoms that emerge within the 3 months after the confirmation of SARS-CoV-2 infection, impair daily functioning and persist for at least 2 months with no other explanation.^1^ Cognitive symptoms are among the most prevalent features of PCC.^2^^,^^3^ People with PCC have now been shown to demonstrate deficits across a wide array of high-level cognitive functions, including sustained attention, cognitive flexibility, and memory.^4^ Such cognitive impairments have been found to correlate with structural and functional brain changes in PCC.^5^

These observations may fit with the general description of their symptoms—“brain fog”—which is commonly used by patients, but there is currently a lack of a robust cognitive signature that distinguishes patients with PCC from those of other people who had SARS-CoV2 infection. This makes it difficult to diagnose with objective markers, and also to develop treatments for cognitive symptoms in this group of patients.

One cognitive abnormality in PCC that has attracted some attention recently is slow processing speed. This has been reported in several investigations conducted in both acute and chronic phases of COVID-19, especially in those with self-reported cognitive symptoms.^6^, ^7^, ^8^, ^9^, ^10^, ^11^, ^12^ However, due to the lack of consensus on the precise definition of PCC^13^^,^^14^ and vast differences in cognitive task design and administration, it remains unclear whether PCC is associated with generalised cognitive slowing.

We aim to test if a fundamental deficit—cognitive slowing (here defined as increased time to process information and respond to it)—is present in people with PCC. We compared patients with PCC to healthy people who previously contracted COVID-19 but did not experience PCC, as well as a second control group of healthy people who have never contracted COVID-19 before. In a self-administered psychomotor assessment on their own laptop, patients with PCC showed distinctly slower reaction time on a 30-s simple reaction time (SRT) task. Further, performance on this task could predict slowing on a more complex test of sustained attention—the number vigilance test (NVT). Combining the reaction times in these two tasks and questionnaire-derived depression score, predicted with high accuracy whether a previously infected person suffered from PCC.

## Methods

### Participants

194 patients who fulfilled the National Institute for Health and Care Excellence (NICE) criteria for PCC completed this study. They were diagnosed at the post-COVID centre, Department of Internal Medicine and Department of Neurology, Jena University Hospital, Germany (Table 1). For replicating the finding in SRT in the Jena PCC group, we then recruited a second group of patients with PCC (n = 76) diagnosed at Long COVID clinic in Oxford, UK. All patients with PCC completed the study between May 18, 2021 and July 4, 2023 in Jena University Hospital, Jena, Germany and Long COVID clinic, Oxford, UK. All patients’ SARS-CoV2 infection was confirmed by PCR testing more than 12 weeks before testing and between March 1, 2020 and October 7, 2022. Their performance was compared with that of two control groups: The **No-COVID group**, i.e., healthy controls with no COVID-19 history, and the **No-PCC group**, i.e., people who had COVID-19 12 weeks ago but were not experiencing PCC at the time of testing. See Supplementary Methods Section 1*: Participants and demographic characteristics* in Supplementary Materials for more details.

**Table 1:** Participant demographics, questionnaire-derived measures and objective measures of simple reaction task and number vigilance test.

Measure		PCC	No-PCC	No-COVID	PCC vs No-PCC		PCC vs No-COVID		No-PCC vs No-COVID	
					P value	BF10	P value	BF10	P value	BF10
Demographics	n	194	63	113						
	Age	48.8 (10.1)	48.7 (10.5)	48.3 (11.5)	0.92	0.16	0.64	0.14	0.8	0.18
	Gender, female, n (%)	144 (74.2)	36 (57.1)	52 (46.0)	0.01		<0.001		0.16	
	Education, mean years (SD)	10.6 (1.0)	14.8 (2.4)	15.1 (2.5)	<0.001	>1000	<0.001	>1000	0.41	0.25
COVID-19	WHO COVID-19 Severity Scale	2.6 (1.0)	2.1 (0.4)	0	<0.001	>100				
	Time from COVID-19 diagnosis, mean days (SD)	325.7 (155.2)	385.6 (199.2)	n/a	0.014	2.67				
	Stayed at hospital overnight for COVID-19, yes (% of known n)	32 (20.4% of 157)	7 (11.1% of 63)	n/a	0.1					
	Pre-existing neurological or psychiatric conditions, yes (% of known n)	30 (27.3% of 110)	unknown^a^	unknown^a^						
	Concentration difficulty, yes (% of known n)	135 (69.6% of 194)	15 (23.8% of 63)							
Questionnaires	Depression–PHQ-9	10.2 (4.8)	4.7 (5.2)	4.8 (5.5)	<0.001	>1000	<0.001	>1000	0.96	0.19
	Depression–HADS	7.2 (4.7)								
	Anxiety–HADS	7.4 (4.6)								
	Fatigue–FAS	33.4 (8.0)								
	Fatigue–BFI	12.6 (16.9)								
	Sleep quality–PSQI	8.9 (3.4)	7.8 (2.8)	7.7 (2.3)	0.036	1.36	0.028	1.68	0.98	0.19
	Daytime sleepiness–ESS	13.1 (5.0)								
	PTSD	36.7 (12.2)								
	Premorbid IQ–MWT-B	28.9 (4.6)								
Simple reaction time	Mean RT (s)	0.49 (0.20)	0.35 (0.10)	0.33 (0.06)	<0.001	>1000	<0.001	>1000	0.07	0.85
	Age-adjusted RT (z)	3.05 (3.50)	0.59 (2.15)	0.12 (1.37)	<0.001	>100	<0.001	>1000	0.12	0.56
	CV of RTs	0.22 (0.09)	0.25 (0.20)	0.22 (0.13)	0.3	0.34	0.79	0.21	0.34	0.28
	Age-adjusted CV (z)	0.38 (1.13)	0.56 (2.10)	0.31 (1.43)	0.6	0.24	0.78	0.21	0.4	0.25
Number vigilance test	Mean RT (s)	0.61 (0.07)	0.57 (0.08)	0.57 (0.07)	<0.001	>100	<0.001	>1000	0.73	0.18
	Age-adjusted RT (z)	0.76 (1.16)	0.09 (1.39)	0.11 (1.07)	<0.001	>100	<0.001	>1000	0.94	0.17
	Mean accuracy	0.70 (0.20)	0.73 (0.22)	0.78 (0.16)	0.34	0.24	<0.001	36.79	0.09	0.67
	Normalised accuracy (z)	−0.69 (1.41)	−0.54 (1.75)	−0.08 (1.11)	0.48	0.2	<0.001	>100	0.035	1.34
	Normalised change in RT (z)	0.39 (6.59)	−0.14 (1.76)	0.04 (1.12)	0.53	0.19	0.58	0.15	0.42	0.23
	Normalised change in accuracy (z)	0.07 (1.34)	−0.19 (1.28)	−0.09 (1.28)	0.18	0.37	0.32	0.21	0.6	0.19
	On-task tiredness (%)	56.57 (25.03)	50.21 (29.59)	47.33 (26.20)	0.1	0.57	0.003	10.16	0.51	0.21
	Change in tiredness (slope)	0.18 (1.32)	−0.05 (1.48)	0.16 (1.19)	0.24	0.3	0.92	0.13	0.29	0.29

In the Post-COVID condition (PCC) group, all patients met the NICE requirements for PCC. We recruited healthy control participants based on their self-reported health who were healthy and unaware of any neurological conditions. They were then split into two groups based on their responses regarding their history of COVID-19. All participants who self-reported unconfirmed PCC were excluded. All metrics shown in this table are reported as a group mean and 1 standard deviation (SD). T-tests and χ2-tests were used to assess between-group differences, with Bayes Factor 10 reported as well. <0.001 indicates significant P value which passed Bonferroni corrections. WHO: World Health Organization. PHQ-9: Patient Health Questionnaire-9. HADS: Hospital Anxiety and Depression Scale. FAS: Fatigue Assessment Scale. BFI: Brief Fatigue Inventory. PSQI: Pittsburgh Sleep Quality Index. ESS: Epworth Sleepiness Scale Sleep Test Questionnaire. PTSD: Post-Traumatic Stress Disorder Test. MWT-B: Multiple Choice Word Test-B for premorbid intelligence.

a As control participants claimed to be unaware of any neurological or psychiatric conditions, we may assume that they did not have (pre-)existing conditions.

### Ethics

The study was carried out in accordance with the Helsinki II ethics regulations. All participants gave electronic informed consent prior to the experiment. Ethics were approved by the ethics committee of Jena University Hospital (Approval Reference: 5082-02/17) and South Central—Oxford A Research Ethics Committee (Approval Reference: 18/SC/0448).

### Simple reaction time task (SRT)

**Fig. 1:**
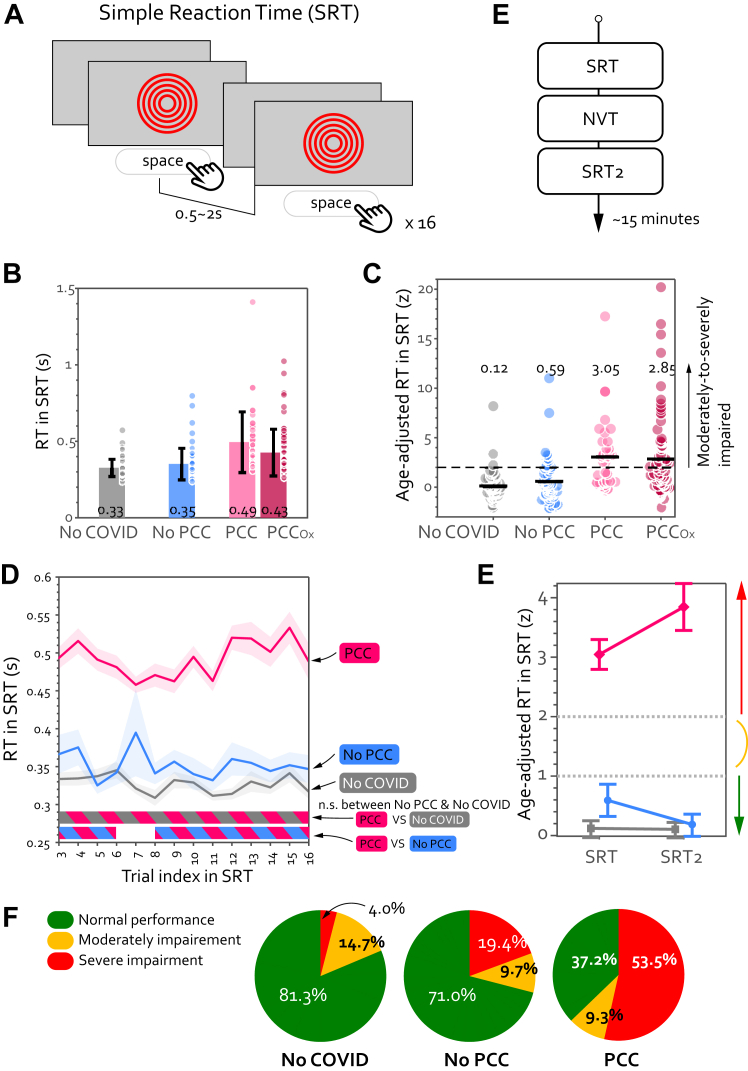
**Patients with PCC were slower than people without PCC, including those who had previously contracted COVID-19.** (A) Simple Reaction Time (SRT) task contained a total of 16 trials. The mean RT for each group is shown in (B) with each dot representing individual data and the error bar showing ± 1 SD. Except for the first two trials, which were exceptionally noisy and sluggish, the mean RT was calculated from all trials for each participant. PCC Ox indicates the PCC Oxford group. The value at the bottom of each bar indicates the mean of that group. (C) To account for the effect of age on response speed, all participants' speed were adjusted based on the No-COVID controls in the same age. Z-score indicates the number of standard deviations from the age-matched normative population. The coloured circles indicate individual results and the black solid line marks the group mean. The horizontal dash black line indicates the threshold for severe impairment (2 SD). (D) Throughout the SRT, PCC participants (pink curve) reacted significantly slower than the other two groups. RT was computed for every trial in SRT across participants and plotted against the trial index. The shaded area shows ±1 SEM and the horizontal lines at the bottom indicate time intervals where bootstrap statistics confirmed significant differences between two groups (P < 0.05). While there was no difference between No-COVID (grey) and No-PCC (blue), PCC (pink) was significantly slower than No-COVID throughout the SRT task (pink-grey stripy horizontal line at bottom) as well as No-PCC (pink-blue stripy horizontal line). (E) To replicate the result seen in SRT, all participants completed the same task again after the second task. (E) The group mean for both sessions of SRT is plotted with group as separate lines. No-COVID (grey line) and No-PCC (blue line) showed a normal performance in both sessions (below the threshold for moderate impairment (>1 SD). In contrast, PCC (pink line) showed severe impairment (>2 SD) in both sessions. The error bar indicates 1 standard error of mean. (F) On the individual level, most of No-COVID and No-PCC controls had a normal speed (<1 SD) while most of patients with PCC showed significant impairment.

Participants first completed **SRT**, which required them to press the spacebar when a large red circle appeared in the centre of the screen (Fig. 1A). The diameter of the circle was scaled as 50% of the screen height. Once participants pressed the spacebar, the red circle disappeared and would reappear after a randomised time interval between 0.5 and 2 s. There were a total of 16 trials, with the results of the first two trials omitted from further analysis. The mean and coefficient of variance (CV) were then computed for the remaining 14 trials.

This task was performed by 119 patients with PCC (age 46.6 (SD 12.2, range 19–75, 80 females (67.2%)). In addition, 63 No-PCC participants (age 48.7 (10.5), 36 females), and 75 No-COVID participants (age 46.6 (11.9), 29 females) also completed.

### Number vigilance test (NVT)

194 participants completed the NVT, an online visual sustained attention task described in the preceding study.^15^ Participants were required to maintain alertness, monitor a rapidly changing stream of numbers, and press the spacebar when they spotted the uncommon target “0”. Try it in both English and German at [https://octalportal.com/pcc/]. After every minute, participants reported their level of fatigue (“How tired do you feel now?”) and motivation (“How motivated do you feel?”) using a visual analogue scale. Accuracy was computed as true positive rate. Change of reaction time/accuracy was computed as the slope of the first-degree polynomial curve fitted for the 9 min (9 time-points), using MATLAB function polyfit.

Both tasks were implemented using PsychoPy v2021.1.2. and hosted on pavlovia.org. All participants used Chrome browser on a desktop/laptop computer with a keyboard.

See Supplementary Methods Section 2*: Experimental design* in Supplementary Materials for more details about these two tasks and online testing.

### Questionnaires

All participants completed two questionnaires for measuring depression level (Patient Health Questionnaire-9, PHQ-9) and their sleep quality (Pittsburgh Sleep Quality Index, PSQI).

All patients with PCC also completed the following list of questionnaires:

1. Hospital Anxiety and Depression Scale (HADS-D)^16^
2. Fatigue Assessment Scale (FAS)^17^
3. Brief Fatigue Inventory (BFI)^18^
4. Epworth Sleepiness Scale (ESS)^19^
5. Post-traumatic stress-scale-14 (PTSS-14)^20^
6. Mehrfachwahl-Wortschatz-Intelligenztest (MWT-B) for Premorbid IQ^21^

See Supplementary Methods Section 3*: Questionnaires* in Supplementary Materials for more details.

### Statistical analysis

For analysis and data visualisation purposes MATLAB (version R2023a) and R studio (version 12.0) were used.

In the current study, RT strongly positively correlated with age within the No-COVID control group (r = 0.39, P < 0.0001). To take care of the effect of age, z-score (i.e., number of standard deviations from the mean of the normative population in the similar age (±5 yrs) was computed for each variable and each participant, based on the No-COVID control group. Although PCC group had a higher proportion of females, we did not adjust the RT for the gender factor in the present study. We ran a 3 (group: PCC, No-PCC, and No-COVID) x 2 (gender: female and male) ANOVA on RT in SRT. There was no interaction between group and gender (F (2247) = 1.65, P = 0.20), and no main effect of gender (F (1247) = 0.20, P = 0.65).

P values for all group comparisons (t-test for continuous variables or χ2 test for categorical variables) were adjusted with Bonferroni correction. Bayes factor (BF10) was reported when applicable. A non-parametric bootstrap-based statistical analysis was used to identify time intervals in which groups exhibit differences.^22^ All reported bivariate correlations were performed using Pearson's correlation method. Kendall's correlation method is used for correlation between a continuous measure (e.g., reaction time) and an ordinal measure (e.g., WHO COVID-19 severity scale). To investigate the effect of mental health and group on cognitive slowing, the questionnaire-derived mental health metrics were first z-scored across all participants, and generalised linear models (GLM) were conducted in MATLAB, using the function fitglme.

Group classification prediction used MATLAB-based algorithms for the purpose of feature ranking to estimate the absolute contribution of each metric. The fscchi2 function in MATLAB was used to predict group classification and produce the rank's importance scores. All importance scores were then transformed into P values by calculating the exponential of their negative value. Group classification was done through multiple logistic regression using MATLAB function fitglm and its ROC curve was plotted using the MATLAB function perfcurve.

### Role of the funding source

The funder of the study had no role in study design, data collection, data analysis, data interpretation, or writing of the report. All authors had full access to all the data in the study and accept responsibility for the decision to submit for publication.

## Results

### Severe psychomotor slowing in PCC

Participants first completed **SRT** (Fig. 1A). Regardless of prior COVID-19, the average reaction time (RT) for healthy controls (collapsed across No-COVID and No-PCC groups) was 0.34 ± 0.01 s. In contrast, patients with PCC responded significantly more slowly, with a mean of 0.49 s (Table 1; Fig. 1B).

Psychomotor speed prolonged with healthy ageing (RT positively correlated with age amongst controls: Pearson r = 0.27, P = 0.0014). Therefore, we accounted the effect of age for all objective metrics (e.g., RT in seconds) by computing number of standard deviations from the mean of the No-COVID group in the same age group (±3 yrs). The mean RT for patients with PCC was 3.05 ± 0.02 SDs longer than that of their age-matched No-COVID controls (Fig. 1C). This significant delay in response was evident from the start of the SRT (Fig. 1D).

PCC had a normal coefficient of variance in RT (Table 1), indicating that their responses were slow but not more variable. Individuals with recent (re-)infection were excluded, indicating that this considerable rise in RT cannot be solely attributed to (sub-) acute cognitive alteration following the COVID-19 infection.^4^^,^^23^, ^24^, ^25^

We further replicated this finding with the same individuals 10 min later, indexed as SRT2 (Fig. 1D). Patients with PCC were substantially impaired compared to the two control groups in both sessions (Fig. 1E; 2 (session) x 3 (group) ANOVA showed a main effect of group (F (2354) = 49.4, P < 0.001, η2 = 0.22), but no effect of task (F (1354) = 0.13, P = 0.7, η2<0.001). Post-hoc comparisons with Bonferroni correction show PCC > No-PCC (difference = 3.06 ± 0.37 z, t = 8.31, P < 0.001) and PCC > No-COVID (difference = 3.33 ± 0.36, t = 8.2, P < 0.001) regardless of the session.

We define any patient with speed slower than 1 SD from the norm average as moderate slowing and those above 2 SD from the average as severe cognitive slowing. In the current sample, 53.5% of patients with PCC showed severe cognitive slowing, in contrast to 4.0% in No COVID control group (Fig. 1F). Overall, PCC group participants had significantly higher proportion of moderate-to-severe impaired cases than No-PCC group (29.1%, χ2 (1,N = 60) = 11.8, P = 0.0006) and the No-COVID group (18.7%, χ2 (1,N = 77) = 23.5, P < 0.0001).

Out of 194 patients with PCC, 72 completed the Montreal Cognitive Assessment (MoCA) in person. They had an average score of 27.7 ± 0.1, ranging between 21 and 30. 12.6% showed poor global cognition, as they scored below the cognitive-impairment cut-off 26. However, there was no correlation between the MoCA score and their cognitive slowing (Pearson's correlation between MoCA and age-adjusted SRT: r = −0.003, P = 0.98; correlation with raw SRT: r = −0.17, P = 0.16).

Is this cognitive slowing specific to this group of patients with PCC? To answer this question, the same SRT was administered to 76 patients with PCC diagnosed in the Long COVID clinic in Oxford, UK (PCC-Ox group, see Supplementary Materials). This cohort also demonstrated significant RT increases (2.83 ± 0.05 z slower than their age-adjusted No-COVID controls) and did not differ from the patients with PCC tested in Jena (t (116) = 0.29, P = 0.77, BF = 0.21, Fig. 1C). This replication in a separate centre in a different country provides evidence that the psychomotor slowing is generalised among patients with PCC.

### Is cognitive slowing associated with mental health?

**Fig. 2:**
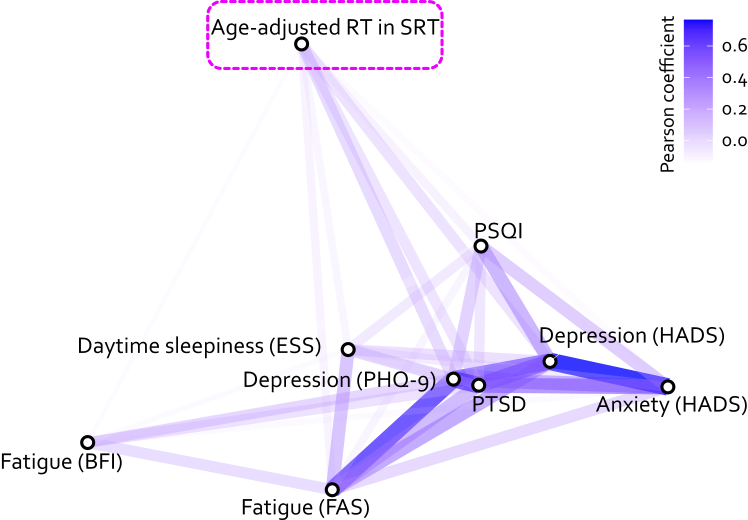
**Network plot of relationships between the cognitive slowing (age-adjusted RT in SRT, highlighted with pink dashed rectangle) and all self-reported metrics (bottom cluster) amongst the patients with PCC.** All depicted relationships are associated with a positive correlation and are rendered in blue. The shorter the distance between two metrics, the stronger their relationship (the higher the correlation coefficient). PHQ-9: Patient Health Questionnaire-9. HADS: Hospital Anxiety and Depression Scale. FAS: Fatigue Assessment Scale. BFI: Brief Fatigue Inventory. PSQI: Pittsburgh Sleep Quality Index. ESS: Epworth Sleepiness Scale Sleep Test Questionnaire. PTSD: Post-Traumatic Stress Disorder Test. Although depression did not predict the cognitive slowing, the combination of depression level and age-adjusted speed in SRT and number vigilance test (NVT) predicts PCC accurately.

Depression and sleep deprivation may increase RT.^26^, ^27^, ^28^ Patients with PCC exhibited moderate to severe depressive symptoms and substantially less restful sleep compared with the two control groups (Table 1), consistent with previous reports of high prevalence of mood and sleep dysregulation after COVID-19 infection.^29^ However, age-adjusted RT in SRT showed no relationship with self-reported depression (PHQ-9 and HADS), anxiety (HADS), fatigue (FAS and BFI), sleep disturbance (PSQI and ESS), or post-traumatic stress disorder (PTSS-14) (all r values < 0.27, P values > 0.087). The relationships between cognitive slowing and all questionnaire-derived mental phenotypes are visualised as a network plot in Fig. 2, in which the strength of the relationship is represented by the distance between the metrics. RT, located at the top of Fig. 2, showed a clear dissociation from the mental health phenotypes which were closely related to each other, clustering on the right side of the figure.

This dissociation between cognitive slowing in PCC and mental health symptoms is further supported by a GLM analysis. The GLM examined the relationship of cognitive slowing to the level of depression and the diagnosis (PCC or not) amongst all participants (age-adjusted RT ∼ PHQ-9∗group). The results of this analysis revealed a significant main effect of group [F (1157) = 18.26, P < 0.0001], but no interaction between group and depression [F (1157) = 1.83, P = 0.18] and no effect of depression [F (1157) = 0.25, P = 0.62]. Similarly, no relationship was found with sleep disturbance level (age-adjusted RT ∼ PSQI ∗ group; no interaction [F (1160) = 0.47, P = 0.49], no main effect of PSQI [F (1160) = 0.34, P = 0.56] but significant effect of group [F (1160) = 29.49, P < 0.0001]).

### Patients with PCC were slow and less vigilant

**Fig. 3:**
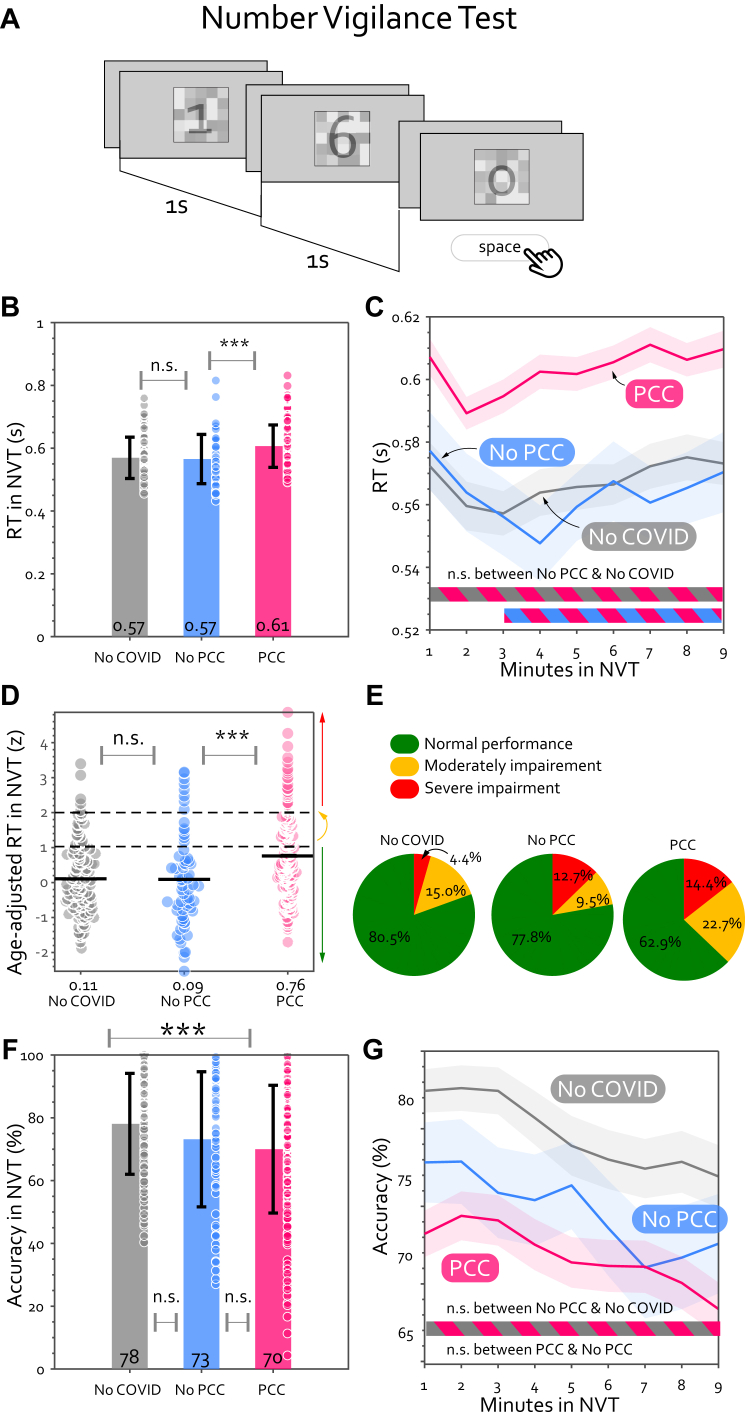
**Patients with PCC responded slower and worse at detecting targets in the Number Vigilance Test (NVT).** (A) Following the SRT, all participants completed the NVT, in which a number between 0 and 9 was displayed at 1 Hz and it was displayed for 0.1 s while being masked by a transparent checkerboard. Participants were instructed not to press anything if the number was between 1 and 9 and press spacebar as soon as possible when seeing the target number 0. The frequency of the target “0” was low (25%) and would not happen consecutively. The mean RT over 9 min for each group is shown in (B), and the mean RT for every minute of this test was plotted against the time (C). n.s. means no significant difference. ∗ means P < 0.05, ∗∗∗ means P < 0.001 and passes multiple comparison corrections. The shaded area shows ±1 SEM and the horizontal lines at the bottom indicate time intervals where bootstrap statistics confirmed significant differences between two groups (P < 0.05). (D) To account for the effect of age on RT in the NVT, all participants' speed were age-adjusted based on the No-COVID controls in the same age. Z-score indicates the number of standard deviations from the age-matched normative population. The coloured circles indicate individual results and the black solid line marks the group mean. The two horizontal dash black lines indicate the thresholds for moderate (>1 SD) and severe impairments (>2 SD). (E) The pie charts show the proportion of individuals who had a normal speed (<1 SD, green area), moderate impairment (>1 SD, yellow area) and severe impairment (>2 SD, red area) in this test. The mean accuracy during this task is plotted in (F) and against time in (G). PCC (pink) was significantly less vigilant than No-COVID throughout the SRT task (pink-grey stripy horizontal line at bottom).

Next, we asked if the PCC slowing is also present in a cognitively more demanding task than the SRT. To investigate this, we used NVT, a paced vigilance test which emphasises that participants should try to be accurate in their responses, with RT being an implicit measure (Fig. 3A). Similar to their slowness on the SRT, patients with PCC took substantially longer to react to targets compared to healthy controls (Fig. 3B), regardless of whether the controls had COVID-19 previously or not (Table 1). The slowness was maintained throughout the entire course of 9 min (Fig. 3C). Importantly, RT on the NVT was strongly associated with the slowness observed in SRT, as age-adjusted RT in SRT alone could explain 35.4% of variance in age-adjusted RT in NVT amongst patients with PCC (F (1179) = 30.7, P < 0.001). Overall, patients with PCC showed mild, yet significant, cognitive slowing (Fig. 3D) and a smaller proportion of patients with PCC showed severe impairments in this task (i.e., people whose speed was slower than 3 SD from the healthy controls in their age, Fig. 3E).

Again, cognitive slowing on this task could not be explained by depression (GLM of age-adjusted RT with normalised PHQ-9 score and group; no interaction [F (1271) = 0.24, P = 0.63], no effect of PHQ-9 [F (1271) = 0.0.04, P = 0.84], but significant effect of group [F (1271) = 18.21, P < 0.0001]) or sleep disturbance (GLM with normalised PSQI score and group; no interaction [F (1202) = 0.55, P = 0.46], no effect of PSQI [F (1202) = 0.06, P = 0.80], but significant effect of group [F (1202) = 39.45, P < 0.0001].

In addition to slowing, patients with PCC were also less vigilant to visual stimuli compared to the uninfected participants (Fig. 3D, t (305) = −3.95, P < 0.0001, BF10 = 194.7). However, the vigilance level could not distinguish if an infected individual was experiencing PCC or not (no significant difference between PCC and No-PCC groups: t (255) = −0.95, P = 0.34 BF10 = 0.24; Fig. 3F). Patients with PCC also showed good maintenance of vigilance over time (Fig. 3G). Although their accuracy significantly declined over time (t (193) = −3.5, P < 0.001, BF10 = 25.3), the decrement was minimal in magnitude (slope of accuracy over time = −0.005 ± 0.002 min^−1^), statistically similar to the No-PCC and No-COVID controls (Table 1).

**Fig. 4:**
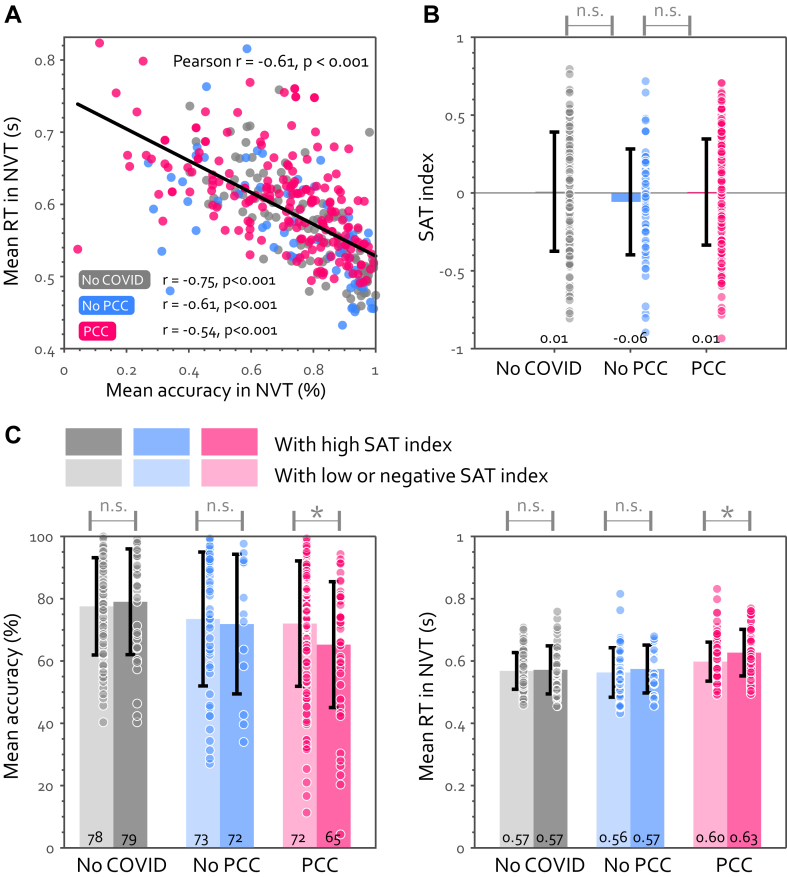
**The slowness in PCC cannot be explained by speed-accuracy trade-off (SAT).** (A) RT is negatively correlated with accuracy in the NVT across participants in every group. Pearson's r and P values for all participants are shown at the top right and those for each group are shown at the bottom left of the graph. The SAT index for each group is shown in (B) with each dot representing individual data and the error bar showing ± 1 SD. SAT index for every participant is computed as the correlation coefficient of RT and accuracy for every minute. On the group level, PCC didn't show any difference in the tendency to employ SAT in this test, as the SAT index is not significant above zero. (C) Comparing the objective performance within each group, between participants with high SAT index (i.e., median split of SAT index, shown in the right-hand side dark colour bars) and the rest. n.s. means no significant difference. ∗ means P < 0.05.

Could patients with PCC deliberately slow down to maintain accuracy (aka, speed-accuracy trade-off, SAT)? The results showed the converse: RT negatively correlated with accuracy (Fig. 4A, r ≤ −0.54, P < 0.001 in all three groups), indicating that slower individuals actually had lower vigilance too. This pattern remained true within individuals: patients with PCC with a higher tendency for SAT (i.e., blocks with longer RT associated with higher accuracy, Fig. 4B) were poorer performers overall in both accuracy and speed (Fig. 4C).

One possibility for the good vigilance in PCC is that in order to reach good performance, patients worked harder. To examine this, we asked participants to rate their fatigue after every minute in NVT (“on-task tiredness”). Patients with PCC with normal response speed felt substantially more tired (55.3 ± 2.2%) than other participants with normal speed (46.4 ± 2.2%, t (265) = 2.9, P = 0.004, BF = 6.4), suggesting they found sustaining attention on task more demanding.

### Cognitive slowing and depression distinguish PCC from healthy infected individuals

**Fig. 5:**
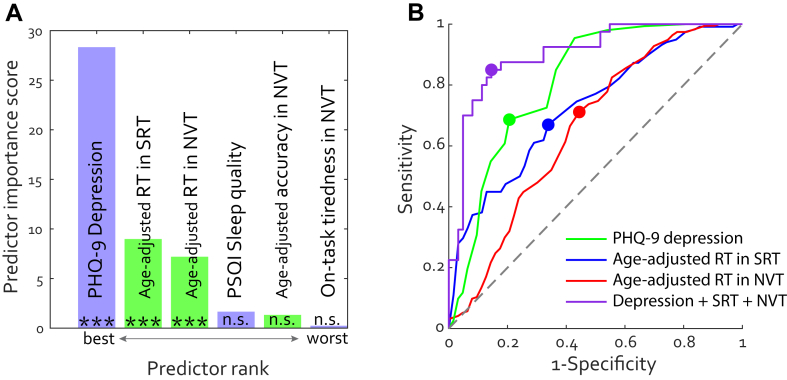
**(A) Ranked measures in predicting group PCC or No-PCC.** Cognitive measures are marked in lime, and self-reported measures (depression, sleep quality and on-task tiredness). ∗∗∗ indicates P values below 0.001. n.s. means not significant. (B) Receiver Operating Characteristics (ROC) curves for group classification. The filled dots indicate the optimal operating points for each model. The purple curve represents the performance of a multiple logistic regression model with all three predictors, PHQ-9 derived depression level, age-adjusted RTs in SRT and NVT.

We then analysed which factors—mental health symptoms and objective cognitive metrics—were most effective in distinguishing infected individuals with PCC from other infected individuals without PCC, i.e., sensitivity for correctly assigning group membership to PCC. We selected all the metrics that showed significant group differences in this study and ranked them according to their importance in predicting PCC in infected individuals (i.e., PCC or No-PCC, Fig. 5A). The rank represents the negative log of the P values. Depression level was the best predictor, followed by RT in the SRT and in NVT. These three were significant predictors of the group (all P < 0.0001), while PSQI and other key metrics in the NVT were not.

Fig. 5B depicts the performance of these three significant predictors as ROC curves. PHQ-9 deprived depression scale (green curve) had a strong Area Under Curve (AUC) of 0.81, with 68.6% sensitivity and 79.4% specificity, at a cut-off score of 7 (i.e., if individuals scored PHQ-9 above 7). SRT (age-adjusted RT) could fairly distinguish individuals with PCC from those without PCC with 0.72 AUC, 66.9% sensitivity, and 66.1% specificity at the cut-off of 0.60 z (i.e., if individuals responded 0.6 SD slower than norm in SRT). NVT alone had slightly lower predicting performance (0.66 AUC, 71.1% sensitivity, and 55.6% specificity). We then used a multiple logistic regression with all three significant variables to classify PCC or No-PCC, which yielded an excellent AUC (0.90) and good sensitivity (85.0%) and specificity (85.5%) (purple curve in Fig. 5B).

### Is cognitive slowing related to severity and time elapsed after acute COVID-19?

Importantly, patients with PCC without being hospitalised due to COVID-19 also showed significant cognitive slowing compared to non-hospitalised No-PCC participants (t (83) = 3.69, P < 0.0001, BF10 = 66.6). This indicates that the cognitive slowing observed in PCC was not merely due to the acute illness of COVID-19.

**Fig. 6:**
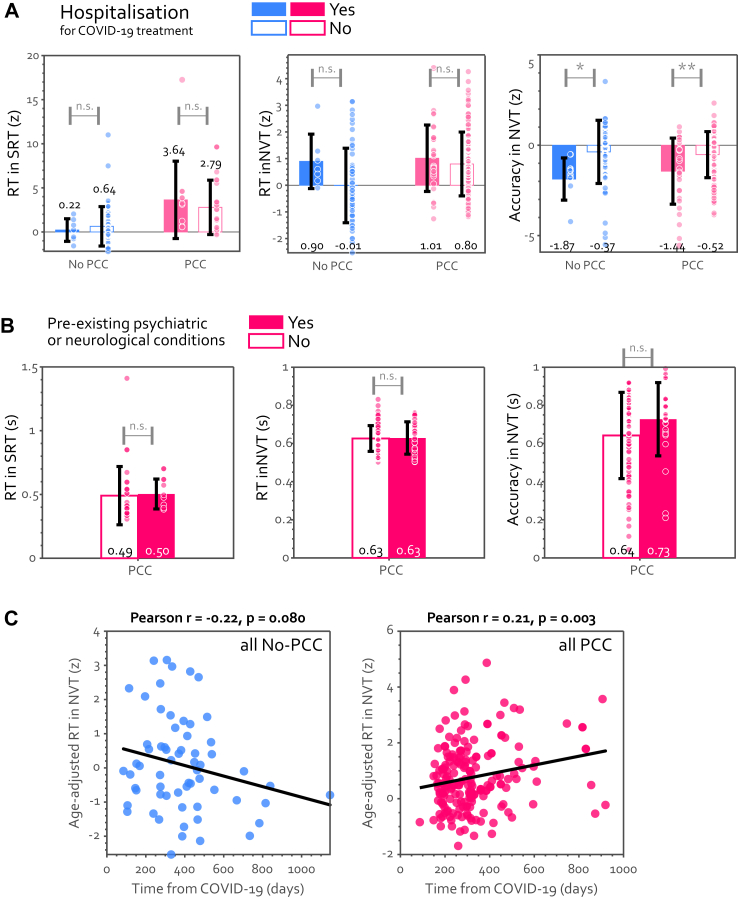
**How did the acute COVID-19 infection affect objective performance?** (A) Although hospitalised individuals, regardless of PCC status, demonstrated no difference in RT in SRT (left) or NVT (middle), they were significantly less accurate (right). N (No PCC inpatients) = 7, N (No PCC outpatients) = 56, N (PCC inpatients) = 32, N (PCC outpatients) = 125. (B) No difference was found between patients with PCC with and without pre-existing psychiatric conditions prior to COVID-19. N (PCC with pre-existing conditions) = 31, N (PCC without pre-existing conditions) = 80. (C) In NVT, age-adjusted RT was marginally negatively correlated with time from infection in No-PCC participants, implying a gradual recovery (left). Cognitive slowing, however, was strongly positively correlated with time from infection in PCC group. n.s. means no significant difference. ∗ means P < 0.05, and ∗∗ means P < 0.01.

Fig. 6A demonstrates that patients with PCC hospitalised due to COVID-19 showed significantly lower accuracy in the NVT but no difference in RT in either task. Even after removing the cases with ICU admission (n = 4), the WHO severity scale remained significantly negatively correlated with the mean accuracy in NVT (Kendall r = −0.18, P = 0.007).

Pre-existing psychological or neurological conditions did not differentiate patients with PCC on objective performance neither (Fig. 6B). Given that depression was the most prevalent of the pre-existing conditions here (45%, see Supplementary Materials), this result is consistent with the absence of the aforementioned relationship between depression and cognitive slowing in PCC.

Does the cognitive impairment get better with time? Patients with PCC showed the reverse trend: prolonged duration of PCC was linked with more severe cognitive slowing (r = 0.21, P = 0.003, Fig. 6C).

## Discussion

The present study reported a significant psychomotor slowing in individuals diagnosed with PCC. Importantly, this cannot be attributed to poor global cognition as measured by a cognitive screening test (MoCA), fatigue, mental health-related symptoms, or speed-accuracy trade-off. Additionally, the data indicate that this impairment does not improve over time. We also replicated this finding within each individual participant as well as with a separate cohort of patients with PCC diagnosed by a different clinic located in a different country.

The existing body of research on chronic deficits in response speed exhibits significant inconsistencies. One of the earliest studies on post-COVID cognitive deficit suggested a substantial impairment in response speed in the acute or sub-acute stage of COVID-19,^6^ while one investigation reported a modest deficiency only in severe or cognitive cases,^12^ and another online study reported no deficit in response speed in infected individuals with cognitive symptoms.^30^ In addition, Martin et al. found a persistent deficit in perceptual processing speed in patients with PCC with cognitive complaints.^10^^,^^31^

This finding offers an elucidation of the discrepancy by conducting a comparative analysis, for the first time, of the objective performance of individuals infected with and without PCC in relation to uninfected controls. Even though most (71.0%) of No-PCC participants had normal SRT, this group had a higher prevalence (19.4%) of cognitive slowing compared with people without infection (4.0%, Fig. 1E). The prevalence of severe cognitive slowing was even higher for PCC individuals (53%). In accordance with the fact that nearly two-thirds of the studies published up until February 2023 failed to adhere to any recognised deficits of PCC as outlined by authorities such as the NICE, CDC, and WHO,^14^ our finding suggests that the inconsistent findings concerning the persistent psychomotor deficit in post-COVID populations are likely due to heterogeneity in defining PCC in the published studies.

Depression and sleep deprivation may increase reaction time.^26^, ^27^, ^28^ However, our findings indicate that mental health symptoms alone cannot fully account for the cognitive slowing in patients with PCC. This is in line with previous studies that found no correlation between the severity of mental health symptoms and chronic post-COVID cognitive deficit.^15^^,^^32^, ^33^, ^34^ Although depression on its own in PCC cannot explain cognitive slowing, Does this cognitive slowing resolve over time? Accumulating evidence suggests that the majority of individuals recover gradually after a mild-to-moderate COVID-19.^4^ However, the estimation of the duration of recovery is controversial, ranging from recovering within four months to not recovering two years after infection.^4^ Here we found that the cognitive slowing in PCC does not seem to resolve on its own. Instead of a gradual recovery (a negative relationship between time from infection and RT) an opposite trend was present amongst patients with PCC; patients who experienced PCC longer had more severe cognitive slowing. However, we must be cautious about the interpretation of the relationship with time since infection in the cross-sectional data. Specific variants of SARS-CoV-2 may be an important risk factor on cognitive slowing, as self-reported PCC symptoms are more common in the earlier waves before the Omicron variant.^3^^,^^35^ Thus, longitudinal studies with computerised speed tests in both patients with PCC and those without PCC are needed to further confirm the group difference in relationship with time since infection.

Furthermore, it is important to acknowledge the constraints associated with this study. One limitation is that the lack of a comprehensive neuropsychological assessment for both PCC and No-PCC participants. Some people with PCC may experience peripheral neuropathy or joint problems, which could impact their motor response. Another limitation is that our study may exhibit bias towards patients with PCC who have cognitive symptoms. In the current study, 69.6% of patients with PCC reported experiencing concentration difficulty, but the rest might have other cognitive symptoms. However, it has been known that cognitive symptoms are not present in all patients with PCC. Therefore, it is crucial to do a re-test on a larger and more diverse PCC cohort.

Understanding of the underlying mechanisms responsible for the chronic cognitive deficit in PCC is still in its infancy, partly due to the lack of an objective signature in PCC. Here we identify a common cognitive deficit in PCC that can be quantitatively measured with an online platform, with all of our code publicly accessible.

## Contributors

SZ, KF, and MH conceived and planned the experiments. SZ made the website to run the experiment. EMM, PAR, ASr, IU, VK, MR, SB, ASt, MS, EF and KF collected patient data. SZ, EMM, AS, AG collected control data. SZ, EMM, AS, AG and PAR contributed to data management and have directly access and verified the data. AS, IU, VK, MR, SB, AS, MS, EM, KF contributed to project administration. Statistical analyses were done by SZ, with input from EMM, PAR, KF, and MH. SZ, EMM, EF, KF and MH contributed to the interpretation of the results. SZ and MH wrote the manuscript with input from all authors. All authors had full access to all the data in the study and accept responsibility for the decision to submit for publication.

## Data sharing statement

De-identified data supporting this study may be shared based on reasonable written requests to the corresponding author. Access to de-identified data will require a Data Access Agreement and IRB clearance, which will be considered by the institutions who provided the data for this research.

The simple reaction time task and the number vigilance task can be tried online at [https://octalportal.com/pcc]. The source code is shared using a Creative Commons NC-ND 4.0 international licence upon reasonable written request to the corresponding author and publicly available at [https://octalportal.com/pcc].

## Declaration of interests

All authors declare no financial or non-financial competing interests.
